# Evaluation of serum zonulin and occludin levels in obsessive-compulsive disorder and the effect of major depressive disorder comorbidity

**DOI:** 10.3389/fpsyt.2024.1395235

**Published:** 2024-07-23

**Authors:** Sertaç Zengil, Esra Laloğlu

**Affiliations:** ^1^ Department of Psychiatry, University of Health Sciences Erzurum Faculty of Medicine, Erzurum, Türkiye; ^2^ Department of Medical Biochemistry, Ataturk University Faculty of Medicine, Erzurum, Türkiye

**Keywords:** obsessive-compulsive disorder, major depressive disorder, brain-intestinal axis, occludin, zonulin

## Abstract

**Objective:**

The aim of this study is to determine whether the levels of zonulin and occludin, tight junctions (TJ) proteins in the intestinal epithelium, will differ between obsessive compulsive disorder (OCD) patients and healthy controls. We also intended to investigate whether these would vary in OCD patients with and without major depressive disorder (MDD) comorbidity and in comparison with healthy controls.

**Methods:**

Sixty patients diagnosed with OCD and 30 healthy controls were included in the study. The cases were administered the Yale-Brown Obsessive Compulsive Scale (Y-BOCS) and the Hamilton Depression Rating Scale (HDRS). The patients were divided into two subgroups based on their HDRS scores and presence of MDD comorbidity. Zonulin and occludin levels were measured using the ELISA method. The research was carried out between April 2021 and October 2021.

**Results:**

Zonulin and occludin levels were significantly higher in the OCD patient group than in the control group (p<0.001). The levels of both were also significantly higher in the OCD patients with MDD comorbidity (OCD+MDD) compared to those without MDD (OCD-MDD) (p<0.001). Zonulin and occludin levels also rose significantly as disease severity increased in the OCD patient group (respectively; p<0.001, p=0.001). The levels of both increased in line with the severity of depression based on HDRS scores in the OCD+MDD group (p<0.001). A positive correlation was determined between the duration of OCD and zonulin and occludin levels. Evaluation of the entire OCD group revealed a moderate positive correlation between Y-BOCS and HDRS scores and zonulin and occludin.

**Conclusions:**

Zonulin and occludin levels in this research were significantly higher in the patients with OCD than in the healthy controls. That elevation was positively correlated with disease duration and severity, and the increase was significantly more pronounced in OCD with MDD comorbidity. These findings point to a possible disorder in the intestinal barrier and blood-brain barrier in OCD patients.

## 1 Introduction

OCD is a common, typically persistent disorder marked by intrusive and disturbing thoughts (obsessions) and repetitive behaviors (compulsions) that the person feels driven to perform. Its relatively high lifetime prevalence (approximately 2-3%). It can progress with loss of functionality in all areas. Its potential to cause serious disability during the course of the disease make OCD particularly important (1). OCD is frequently accompanied by other psychiatric diseases, most commonly major depressive disorder (MDD) (2). When evaluated from an etiological perspective, it is known that OCD is associated with many biological, genetic and psychosocial factors. Although antidepressants (especially serotonin reuptake inhibitors), anxiolytics, antipsychotics and cognitive behavioral treatments have been shown to be effective in treatment, it is a fact that 40-60% of patients still do not benefit sufficiently from the treatment. This situation increases interest in the neurobiology of OCD (3, 4). Several factors are thought to be involved in the etiology of OCD, such as functional impairments in structures including the brain’s orbitofrontal cortex, limbic system, basal ganglia, and thalamus, the involvement of neurotransmitters such as dopamine and serotonin and some neuropeptides in that involvement, and the effect of oxidative stress (5). In recent years, there has been increasing evidence pointing to the inflammatory basis of a psychiatric disorder. There are indications that anti-inflammatory cytokines affect both neurohormonal and neurochemical functions in this process. The primary factor leading to a systemic inflammatory response is increased intestinal permeability, also known as leaky gut syndrome. Increasing evidence has recently pointed to the possibility that increased intestinal permeability may play a role in various psychiatric diseases (6). Specifically, a leaky gut permits the translocation into the circulation from the gut of lipopolysaccharides, molecules found in the outer membrane of gram-negative bacteria. Lipopolysaccharides lead to an increase in the release of proinflammatory cytokines by activating various immune cells, and in low-grade systemic inflammation (7). The structures and mechanical properties of the blood brain barrier (BBB) are similar to those of the intestinal barrier (IB) in several terms (8). Permeability of the IB can be affected by several factors, such as psychological stress, oxidative stress, and proinflammatory cytokines, and leads to neuro-inflammation capable of affecting distant organs, including the brain, and the same factors are known to be deleterious to the BBB (9). There is data in the literature that this two-way interaction between the digestive system and the brain may be compromised by changes in the intestinal microbiota, which may cause immune activation and therefore potentially increase the likelihood of various psychiatric symptoms (10).

Intestinal epithelial cells represent the largest interface between the body’s internal and external environments, with a surface area exceeding 200 m2 (11). The principal functions of the IB are to regulate the absorption of nutrients, electrolytes and water from the lumen into the circulation, to prevent the entry of pathogenic micro-organisms from toxic luminal substances, to regulate the molecular exchange between the environment and the host, and to maintain the balance between defense against antigens and the immune system (12). Tight junctions (TJ) between the cells in the IB also regulate the selective permeability of the cell. The TJ complex consists of more than 150 transmembrane proteins, such as occludin, claudin and tricellulin (13). These transmembrane proteins produce a mechanical connection between epithelial cells and are effective in preventing the passage of liquid and solutes from the intestinal lumen to the subepithelial tissue and between cells into the intestinal lumen (14). Occludin is a TJ integral transmembrane protein that was first discovered. Function studies suggest that it is involved in the maintenance and assembly of TJs. This role of occludin is of critical importance in preventing the passage of materials from the intestinal lumen to the subepithelial tissue and between cells into the lumen (15). Elevated occludin levels in serum have been described as a reliable indicator of IB damage (16).

Zonulin, the only known modulator of TJs, plays an important role in maintaining epithelial barrier and IB tightness (17). It was discovered as a pre-haptoglobulin2 protein via proteomic analysis of human serum. Pre-haptoglobulin 2 (zonulin) was initially thought to be the precursor of haptoglobulin-2, solely responsible for the maintenance of hemoglobin stability, although it was gradually found to exhibit a significant effect on intestinal permeability (18). In its single chain form, zonulin regulates intestinal permeability by stimulating epidermal growth factor receptors, while in its cleaved double chain form, it has been shown to protect hemoglobin against oxidation (17). It binds to specific receptors on the epithelial surface in the intestine, thus binding actin fibers to one another within the cell via protein kinase C and regulates TJ functions. The intercellular junctions open rapidly and reversibly with the release of zonulin under different effects (19). Increased zonulin secretion (levels) indicate increased intestinal permeability, showing that the TJs are permeable rather than closed (17).

Many innovative studies conducted in recent years have highlighted the possible relationship between IB and BBB permeability and psychiatric disorders such as MDD, schizophrenia, autism spectrum disorder, attention deficit hyperactivity disorder and bipolar disorder (20–24). However, the number of studies examining the association between OCD and IB and BBB permeability is very limited (25). Although there are a limited number of studies evaluating zonulin levels in OCD, we did not find any study investigating the relationship between occludin and OCD. However, the number of studies examining the association between OCD and IB and BBB permeability is very limited (25). Although there are a limited number of studies evaluating zonulin levels in OCD, we did not find any study investigating the relationship between occludin and OCD. However, there are limited studies in the literature on claudin-5, a molecule similar to occludin. In the literature, there is data from of studies conducted with pediatric and adult OCD patients that zonulin levels do not change but claudin-5 levels increase (25, 26). The role of IB and BBB dysfunction in the pathogenesis of OCD still remains unclear. The aim of this study is to investigate whether the levels of zonulin and occludin, tight junction proteins in the intestinal epithelium, change in obsessive-compulsive disorder (OCD) patients and whether they differ between OCD patients with and without MDD.

## 2 Methods

Ethical approval for the study was granted by the Erzurum Regional Training and Research Hospital Ethical Committee, Türkiye (Erzurum BEAH KAEK 2021/06-127). The research was carried out between April 2021 and October 2021 at the Department of Psychiatry at Erzurum Regional Training and Research Hospital and the Department of Biochemistry at Atatürk University Faculty of Medicine. It was performed in compliance with the principles of the Declaration of Helsinki, and informed consent was obtained before enrollment from all patients and controls.

### 2.1 Sample size

A minimum total sample of 36, 12 in each group, was calculated at a type 1 error value (alpha) of 0.05 for the zonulin variable, a test power (1-beta) of 0.95, and an effect size of 1.58 based on reports in Hongyan et al.’s research (27). Thirty healthy controls and 60 patients with OCD were included in the study in order to be able to classify the patients among themselves in terms of presence or absence of accompanying depression. To determine whether the sample size was large enough for the analyses, we ran an *a priori* power analysis using the “G*Power 3.1” (28).

### 2.2 Patient group

Sixty patients presenting to the Health Sciences University Erzurum Medical Faculty Mental Health and Diseases Clinic and diagnosed with OCD on the basis of the Diagnostic and Statistical Manual of Mental Disorders, Fifth Edition (DSM-5) criteria were included in the study. None of the patients had used any medication or received any treatment during the previous month. The patients were screened for the presence of MDD on the basis of DSM-5 and were divided into two subgroups based on those results – with accompanying MDD (OCD+MDD) and without accompanying MDD (OCD-MDD).

### 2.3 Control group

The controls consisted of 30 people who applied to Erzurum Medical Faculty of Health Sciences University Psychiatry Clinic and were evaluated as mentally and physically healthy. The healthy controls enrolled in the study were matched with the patient group in terms of age and sex. Individuals with alcohol or substance abuse, chronic systemic or neurological diseases, acute or chronic inflammation, or autoimmune diseases were excluded from the study since these might affect the findings.

### 2.4 Data collection tools

#### 2.4.1 The structured clinical interview for DSM-5 disorders—clinician version

This was employed to investigate psychiatric disorders based on DSM-5. This semi-structured interview guideline was developed by First et al., principally for establishing DSM-5 diagnoses (29). Its Turkish-language validity and reliability were studied by Elbir et al. (30).

#### 2.4.2 Sociodemographic data form

This was employed to investigate the participants’ sociodemographic and clinical characteristics and histories. A sociodemographic and clinical data form prepared by us, in accordance with clinical experience and information obtained from scanned sources, and taking into account the objectives of the study, was used in all cases. This form; it is a semi-structured form that includes sociodemographic information such as age, marital status, educational status, occupation, gender, place of residence, economic status, and clinical data such as age at onset of the disease, duration of the disease, number of hospitalizations, and treatments received during the disease.

#### 2.4.3 Yale-brown obsessive-compulsive scale

The scale is a semi-structured scale administered by a clinician, developed to measure the type and severity of obsessive-compulsive symptoms in patients diagnosed with OCD. This scale, developed by Goodman et al. (31, 32). Turkish adaptation was made by Karamustafalioglu et al. (33). The YBOCS we used in our study consists of 10 items. 1st-5th of these items evaluate obsessions, and 6st-10st evaluate compulsions. The items indicate the severity of the obsession and compulsion. On scale, obsessions and compulsions are scored separately with five items each, each item out of four points. At the end a total score out of a maximum of 40 points is obtained. Intraclass correlation coefficients were found to be 0.88 for each scale. The subscales show a high and significant relationship with each other. Cronbach Alpha reliability coefficient was found to be 0.96, 0.94, 0.93, 0.95, 0.95 and 0.94 for the sub-dimensions. The divergent validity of the scale was found to be satisfactory.

#### 2.4.4 Hamilton depression rating scale

The HDRS was used to confirm the severity of depressive symptoms and well-being during remission. The semi-structured scale includes 17 questions. In the test administered by the clinician, each question is scored between 0 and 4. The maximum score is 51 points. The items indicate the severity of the depression. The scale is administered by the interviewer and was developed by Hamilton (34). Its Turkish-language validity and reliability were studied by Akdemir et al. (35). The Cronbach’s alpha internal consistency coefficient of HDRS over 17 items was found to be 0.75 and the reliability coefficient according to the Spearman-Brown formula was found to be 0.76.

### 2.5 Blood specimens and analyte assay techniques

Blood specimens collected from the OCD patients and control group were centrifuged for 15 min at 4,000 rpm for serum separation for biochemical analysis. The sera obtained were stored at -80°C in Eppendorf tubes until the day of use.

Zonulin and occludin levels were measured with the ELISA method using a Human Zonulin ELISA kit (Catalog No. EEL-H5560; Elabscience Biotechnology Inc., Houston, TX, USA) and a Human Occludin ELISA kit (Catalog No. E-ELH1073; Elabscience Biotechnology Inc.) in line with the manufacturer’s instructions. Kit measurement ranges for zonulin and occludin were 0.78-50 ng/mL and 0.16-10 ng/mL, respectively. The intra-assay coefficient of variation (CV) was 4.9%, with an inter-assay CV of 5.2% for zonulin. The intra-assay CV was 4.8%, with an inter-assay CV of 4.8% for occludin. The costs of the biochemical analyses were met from the researchers’ own budget.

### 2.6 Statistical analysis

Data recording and analysis were performed on SPSS version 20.0 for Windows software (SPSS Inc.). Descriptive statistics were expressed as number and percentage for categorical variables and as mean ± SD for numerical variables. Normality of distribution was evaluated using visual (histograms and probability plots) and analytical methods (the Kolmogorov-Smirnov/Shapiro–Wilk tests). One‐way analysis of variance (ANOVA) and the unpaired Student t-test were used to compare the study groups, and the degree of significance between groups was determined using the *post-hoc* Tukey test. Correlation analyses were performed using Pearson’s correlation test. P values <0.05 were regarded as statistically significant.

## 3 Results

The clinical and sociodemographic characteristics of the patients and controls are shown in **Table 1**. No significant age or gender differences were observed among the groups (p=0.76 and p=0.09, respectively).

**Table 1:** A comparison of the groups’ sociodemographic and clinical characteristics.

	n	%	mean (SD)
**Patients**	60		
Gender			
male	24	40	
female	36	60	
Age, years			32.18 (8,87)
Duration of illness, years			6.52 (4.14)
Subgroups			
OCD + MDD	30	50	
OCD – MDD	30	50	
Scale scores			
HDRS	30		25.9 (5.62)
Y-BOCS	60		21.15 (8,75)
OCD + MDD group according to HDRS			
Moderate depression	16	53.3	
Severe depression	14	46.7	
According to Y-BOCS			
Mild- Moderate OCD	33	55	
Severe OCD	27	45	
**Controls**		30	
Gender			
male	13	43.3	
female	17	56.7	
Age, years			35.50 (8.65)

OCD, Obsessive-Compulsive Disorder; MDD, Major Depressive Disorder; HDRS, Hamilton Depression Rating Scale; Y-BOCS, Yale-Brown Obsessive-Compulsive Scale.

**Figure 1:**
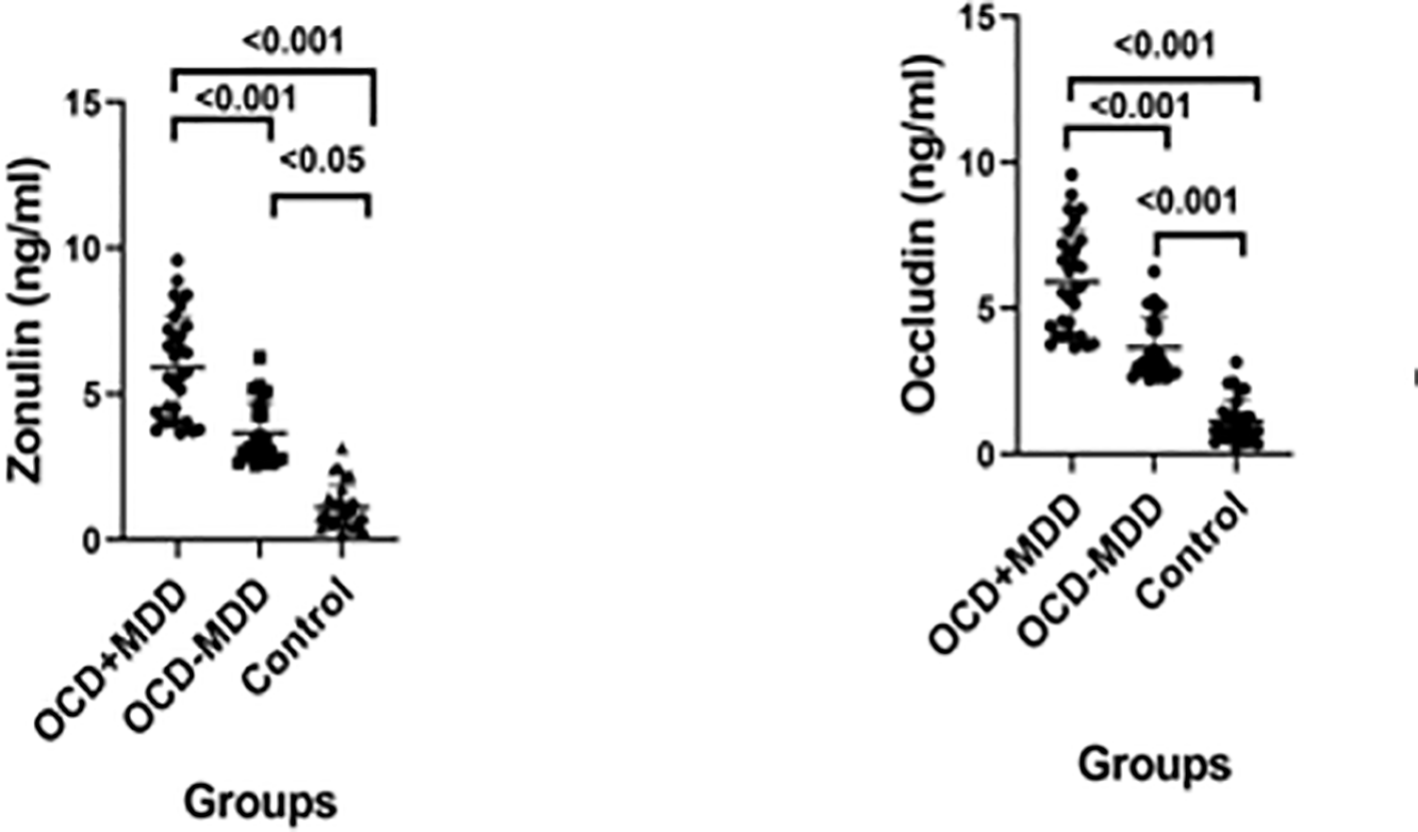
Dot Plots Showing The Goups’ Serum Zonulin and Occludin Levels.

The patients with OCD were divided into subgroups, with MDD comorbidity (OCD+MDD) and without MDD comorbidity (OCD-MDD). The group’ zonulin and occludin levels are shown in **Table 2**. Dot plots showing the statistical significance of serum zonulin and occlusion levels in each diagnostic group presented in **Table 2** are shown in **Figure 1**. The patients registered significantly higher zonulin and occludin levels than the control group. The levels of both proteins were also significantly higher in the OCD+MDD patient group than in the OCD-MDD group (**Table 2**). The OCD patient group was also divided into two subgroups on the basis of their gender. Serum zonulin levels were 18.93 ± 8.44 ng/ml in cases of female group,17.54 ± 8.28 ng/ml in the male gender group. Serum occludin levels were 3.69 ± 2.34 ng/ml in cases of female group,3.37 ± 2.30 ng/ml in the male gender group. No significant zonulin and occludin differences were observed among the female and male genders (p=0.433, p=0.507, respectively). Also in an ANCOVA taking baseline age and gender as covariance the difference between groups remained significant (p<0.001 for both zonulin and occludin).

**Table 2:** The Groups’ Zonulin and Occludin Levels.

	Zonulin (ng/ml)	Occludin (ng/ml)
Groups		
OCD+MDD (n=30)	27.21 (3.47)	5.91 (1.76)
OCD-MDD (n=30)	18.91 (4.05)	3.65 (1.03)
Control (n=30)	8.99 (3.72)	1.12 (0,73)
Statistics		
1 vs. 2 vs. 3^a^	p<0.001	p<0.001
1 vs. 2^a^	p<0.001	p<0.001
1 vs. 3^a^	p<0.001	p<0.001
2 vs. 3^a^	P=0.001	p<0.001

a ANOVA with Tukey’s HSD. Values expressed as mean (SD).

OCD, Obsessive-Compulsive Disorder; MDD, Major Depressive Disorder.

**Figure 2:**
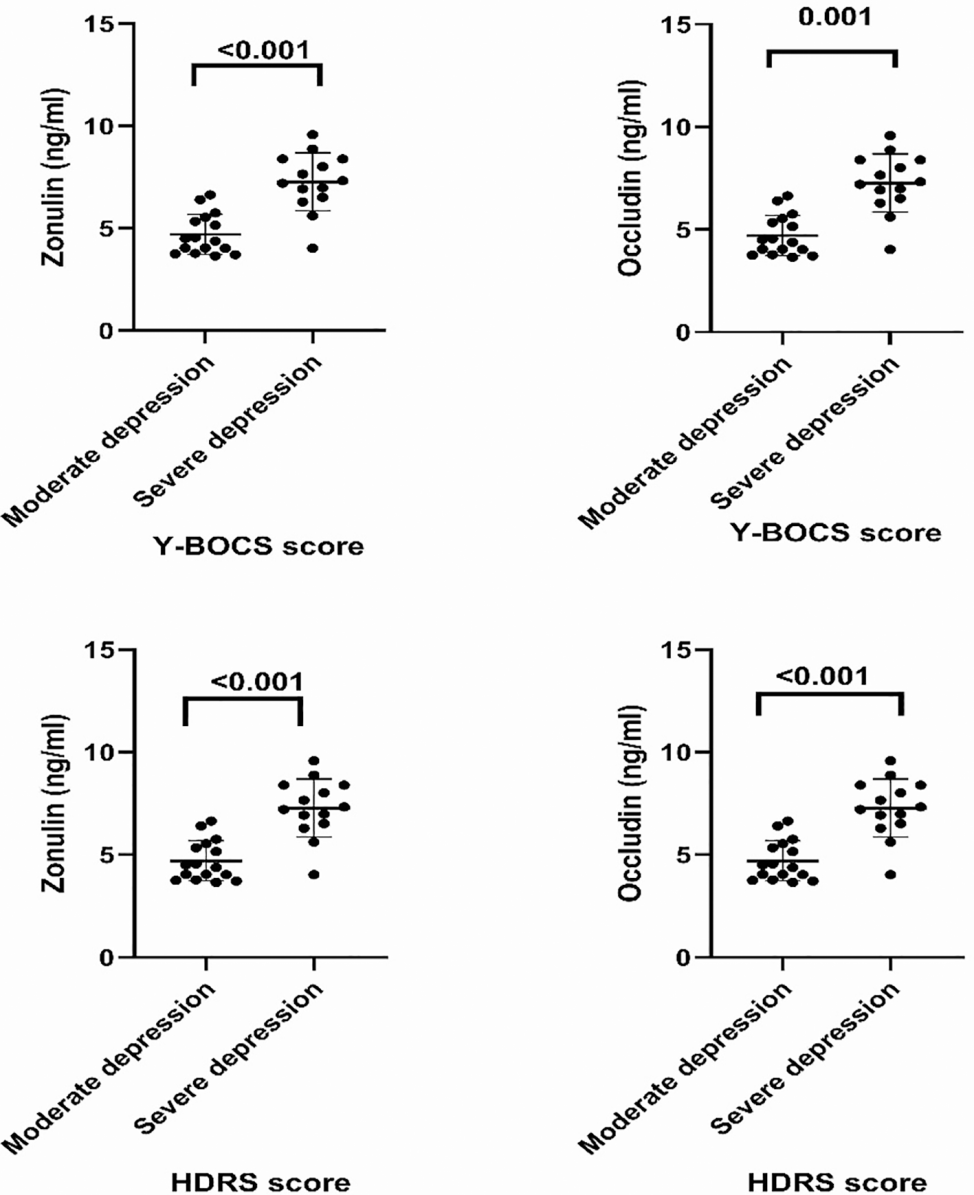
Dot Plots Showing The Goups’ Serum Zonulin and Occludin Levels According to Y-BOCS and HDRS Scores.

The OCD patient group was also divided into two subgroups on the basis of their Y-BOCS scores, mild-moderate and severe. Interestingly, zonulin and occludin levels in the patients with OCD rose significantly in line with the severity of the disease. The OCD+MDD patient group was also divided into moderate and severe subgroups on the basis of HDRS scores. Zonulin and occludin levels also increased in line with the severity of the accompanying depression (**Table 3**). Dot plots showing the statistical significance of serum zonulin and occlusion levels according to the Y-BOCS and HDRS scores presented in **Table 3** are shown in **Figure 2**.

**Table 3:** Patient Group Zonulin and Occludin Levels According To Y-BOCS and HDRS Scores.

	Zonulin (ng/ml)	Occludin (ng/ml)
Groups (Y-BOCS score)		
Mild- moderate (n=33)	20.29 (5.23)	4.09 (1.72)
Severe (n=27)	26.45 (4.02)	5.62 (1.59)
Statistics 1 vs. 2	p<0.001	P=0.001
Groups (HDRS score)		
Moderate depression (n=16)	24.83 (2.40)	4.71 (0.98)
Severe depression (n=14)	29.93 (2.32)	7.27 (1.42)
Statistics 1 vs. 2	p<0.001	p<0.001

a ANOVA with Tukey’s HSD. Values expressed as mean (SD).

HDRS, Hamilton Depression Rating Scale; Y-BOCS, Yale-Brown Obsessive-Compulsive Scale.

**Figure 3:**
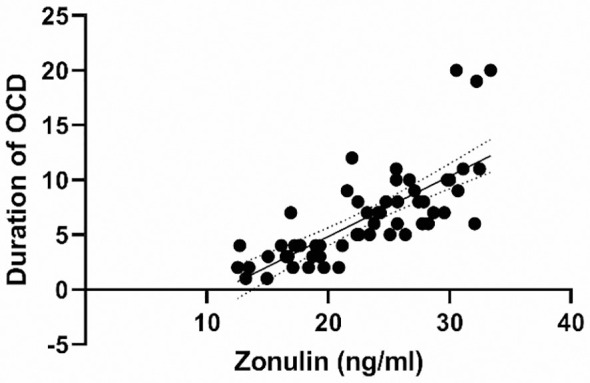
Scatter-Plots Between Serum Zonulin Level and OCD Duration.

A powerful positive correlation was determined between the duration of OCD and both zonulin and occludin (r=0.747 and r=0.711, respectively, p<0.001 for both). Zonulin emerged as the marker with the greatest association with disease duration. 2D scatter plots between serum zonulin level and OCD duration are shown in **Figure 3**. There was no significant correlation between age and serum zonulin and occludin levels in the patient and control groups.

**Figure 4:**
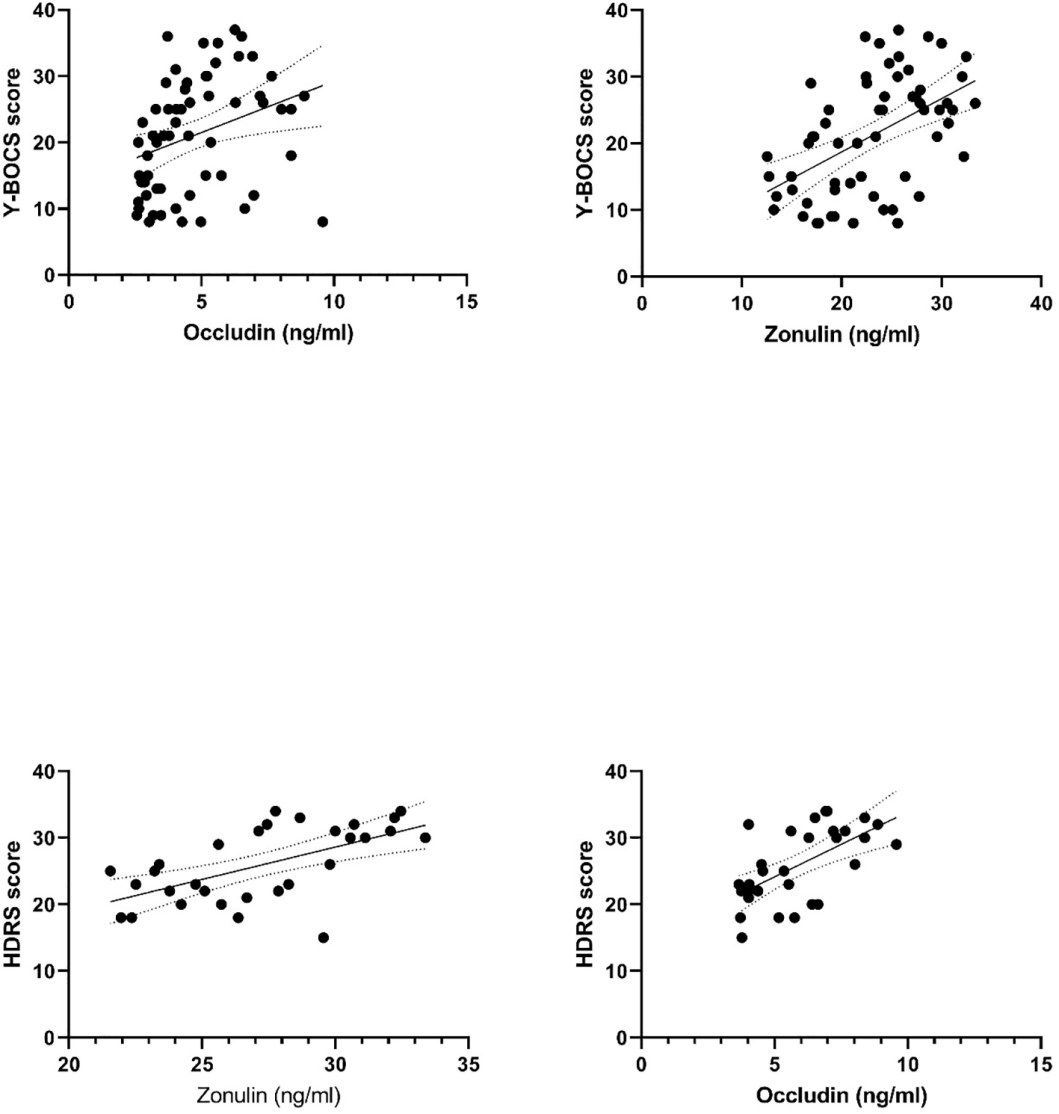
Scatter-Plots Between Serum Zonulin and Occludin Levels and Y-BOCS and HDRS Scores.

Analysis of the total OCD group revealed moderate positive correlations between Y-BOCS and HDRS scores and zonulin and occludin (r=0.514 and r=0.623, respectively for Y-BOBS scores, p <0.001 for both; r=0.600 and r=0.609 for HDRS scores, respectively, p <0.001 for both). Scatter plots between serum zonulin and occludin levels and Y-BOCS and HDRS scores are shown in **Figure 4**.

## 4 Discussion

This study was performed to determine whether serum zonulin and occludin levels in patients with OCD would differ from those of healthy controls and to clarify how these are affected by MDD comorbidity. The results revealed significantly zonulin and occludin levels in the OCD patients, irrespective of age and sex than in the controls. This finding was consistent with our study hypothesis. Although a limited number of studies have evaluated zonulin levels in OCD, we encountered none investigating the relationship between occludin and OCD. This study is thus original from that perspective. Isik et al. observed no difference in zonulin levels between pediatric OCD patients and a control group, but concluded that plasma levels of claudin-5, like occludin an integral protein, were significantly elevated (25). Similarly, in their study of 36 adult patients with OCD, Kilic et al. also reported no difference in zonulin levels compared to a control group, but claudin-5 levels were significantly higher than in the healthy controls (26). Claudin-5 is an integral protein, like occludin, and is responsible for maintaining the health of TJs. Also similarly to occludin, increasing levels of cloudin-5 in plasma are indicative of IB damage (13). From that perspective, claudin-5 levels in both these studies being significantly higher than in the healthy controls is a finding compatible with the significantly higher occludin levels compared to the healthy control group in the present study.

As mentioned in previous sections, zonulin is a modulator of epithelial and endothelial barrier functions. In intestinal dysbiosis, zonulin is released into the circulation through the IB due to intestinal epithelial impairment. Elevated serum zonulin and occludin levels have been shown in the literature to be a reliable parameter indicating IB injury. Such injury is followed by the passage of intestinal contents through the epithelial barrier. This also results in the passage of microbial antigens that trigger T cell activation through the epithelial barrier. This entails an increasing release of proinflammatory cytokines and intestinal permeability. This event represents the beginning of a process that will continue as a vicious circle (13). Proinflammatory cytokines increase during this process and can easily pass through the BBB and affect brain cells, neurotransmitter metabolism, and the hypothalamus-pituitary-adrenal axis (36). Several studies have shown an association between proinflammatory cytokines and OCD (37). DSM-5 distinguishes between primary and secondary forms of organic OCD (38). The etiology of the primary, idiopathic forms of OCD is multifactorial, involving biological, psychological, and external factors. Biological processes include serotonergic, glutaminergic, and dopaminergic neurotransmission irregularity, imbalance in cortico-striato-cortical circuits, genetics and epigenetics, and autoimmune OCD (39). Zonulin is a potential inflammatory marker and has an important role in gut immunity. There are studies showing an increase in IP with inflammation in mental disorders. These data suggest that the significantly higher levels of zonulin and occludin in our OCD patient group compared to healthy controls may indicate an autoimmune form of OCD. However, in light of our knowledge so far, this situation is still at the level of probability.

When the literature was examined, it was seen that zonulin levels were also examined in various psychiatric disorders. Cakir et al. detected higher zonulin and occludin levels in pediatric patients with attention deficit hyperactivity disorder compared to a control group (40). Kilic et al. reported higher zonulin and claudin-5 levels in cases of bipolar disorder than in healthy controls and suggested that this indicated the involvement of intestinal and BBB permeability in the pathogenesis of bipolar disorder (41). Ozyurt et al. determined higher zonulin levels in children with attention deficit hyperactivity disorder compared to a control group (23). In their study examining the relationship between zonulin and autism spectrum disorder, Esnafoglu et al. detected higher zonulin levels in patients with the condition than in a control group (22). Finally, Barber et al. examined serum zonulin levels in 99 cases diagnosed with schizophrenia and concluded that zonulin levels exceeded threshold values in 42 patients (42).

Powerful, positive correlations were observed in the present study between zonulin and occludin and the duration of OCD (that correlation being more marked in the case of zonulin). This data may point to a possible relationship that the persistence of IB permeability may also contribute to the persistence of OCD. However, it is obvious that pharmacologic agents used in the treatment of OCD have effects on appetite and therefore on nutrition. For this reason, mentioning this causal relationship may be a misleading comment made too early for now.

As it is known, two-thirds of OCD patients may have depressive symptoms that meet the diagnostic criteria for major depression (43). In order to investigate the effect of MDD comorbidity on our patient group’s zonulin and occludin levels we divided these into OCD+MDD and OCD-MDD subgroups. Both zonulin and occludin levels were significantly higher in the OCD+MDD patient group than in the OCD-MDD group. We encountered no other studies referring to this comorbidity. Our study is thus also original from that perspective. Wu et al. reported significantly higher plasma levels of zonulin, claudin-5, intestinal fatty acid binding protein, and lipopolysaccharide in adolescents diagnosed with MDD compared to healthy controls (25). However, Maget et al. detected no difference in zonulin levels between their MDD patient group and healthy controls (44). Ohlosson et al. detected higher intestinal fatty acid binding protein and lower zonulin levels in an MDD patient group compared to healthy controls (45). Several lines of evidence support the association between major depressive disorder (MDD) and inflammation. The literature also shows bidirectional interaction pathways between depression and inflammation (46). Intestinal microbiota dysbiosis observed in MDD results in endotoxemia and an inflammatory state, ultimately exacerbating depressive symptoms, while the manipulation of such microbiota by means of prebiotics may help alleviate the severity of depression (47). Consistent with the previous literature, zonulin and occludin levels increased in line with the severity of depression in MDD accompanying OCD in the present research.

Although care was taken to exclude confounding factors as much as possible in this study, there is nevertheless a possibility that our results were confounded by variables that could not be measured, such as smoking, alcohol use, or dietary habits. Moreover, considering the positive effect of probiotic interventions on the symptoms of intestinal microbiota in OCD, the fact that the intestinal microbiota was not evaluated constitutes an important limitation of this study (48). In addition, the fact that intestinal microbiota was not evaluated represents an important limitation of this study. When selecting the healthy control group in the study, it can be considered as another limitation that it consisted of individuals who applied to a psychiatric clinic, no matter how physically and psychologically healthy they were determined to be. Additionally, this study is a cross-sectional study and therefore the data cannot address causality.

## 5 Conclusion

Zonulin and occludin levels in this study were significantly higher in the patients with OCD than in the healthy controls, that elevation was powerfully correlated with the duration and severity of the disease, and the elevation was also significantly more pronounced in the presence of OCD+MDD comorbidity. This finding points to the possible role of disruption of the intestinal and blood-brain barriers in the pathogenesis of OCD. Although our observation of an increase in zonulin and occludin levels in OCD is not a sufficient finding on its own, it provides information that intestinal permeability may play a role in the pathogenesis of OCD and sheds light on new studies that need to be done on this subject. Further longitudinal studies involving larger groups may yield a different perspective concerning the probable role of increased intestinal permeability in the etiology of OCD. In addition, it will be possible to obtain more descriptive data by taking into account the microbiota and nutritional habits of cases and controls in future studies.

## Data availability statement

The original contributions presented in the study are included in the article/supplementary material. Further inquiries can be directed to the corresponding author.

## Ethics statement

The studies involving humans were approved by Erzurum Regional Training and Research Hospital Ethical Committee, Türkiye (Erzurum BEAH KAEK 2021/06-127). The studies were conducted in accordance with the local legislation and institutional requirements. The participants provided their written informed consent to participate in this study.

## Author contributions

SZ: Conceptualization, Data curation, Formal analysis, Investigation, Methodology, Project administration, Resources, Software, Visualization, Writing – original draft, Writing – review & editing. EL: Formal analysis, Investigation, Methodology, Software, Supervision, Validation, Writing – original draft, Writing – review & editing.
